# Time to Move Upstream? A Framework for Expanding the Focus of Freezing of Gait to Prodromal, Subclinical, and Pre‐FOG Stages

**DOI:** 10.1111/ejn.70721

**Published:** 2026-11-04

**Authors:** Jeffrey M. Hausdorff, Kaylena A. Ehgoetz Martens, Moran Gilat, Jorik Nonnekes, Martina Mancini, J. Lucas McKay, Pieter Ginis, Jamie L. Adams, Jennifer R. Mammen, Erika E. Howe, Simon J. G. Lewis, Fay Horak, Alice Nieuwboer

**Affiliations:** ^1^ Center for the Study of Movement, Cognition, and Mobility Tel Aviv Sourasky University Medical Center ‐ Ichilov Tel Aviv Israel; ^2^ Department of Physical Therapy, Gray Faculty of Medical & Health Sciences, and Sagol School of Neuroscience Tel Aviv University Tel Aviv Israel; ^3^ Rush Alzheimer's Disease Center, Rush University Medical Center, and Department of Orthopaedic Surgery Rush Medical College Chicago Illinois USA; ^4^ Department of Kinesiology and Health Sciences University of Waterloo Waterloo Canada; ^5^ Department of Rehabilitation Sciences Research Group for Neurorehabilitation (eNRGy), KU Leuven Leuven Belgium; ^6^ Department of Rehabilitation, Center of Expertise for Parkinson and Movement Disorders, Donders Institute for Brain, Cognition and Behaviour Radboud University Medical Center Nijmegen the Netherlands; ^7^ Department of Neurology Oregon Health and Science University Portland Oregon USA; ^8^ Department of Biomedical Informatics and Department of Neurology Emory University School of Medicine Atlanta Georgia USA; ^9^ Clario, Part of Thermo Fisher Scientific Philadelphia Pennsylvania USA; ^10^ Department of Neurology University of Rochester Rochester New York USA; ^11^ University of Massachusetts Dartmouth College of Nursing and Health Sciences Dartmouth Massachusetts USA; ^12^ Parkinson’s Disease Research Clinic Macquarie University Brain Institute and Clinic Sydney Australia; ^13^ School of Biomedical Engineering Tel Aviv University Tel Aviv Israel

**Keywords:** digital phenotyping, gait instability, motor–cognitive interactions, Parkinson's disease, wearable sensors

## Abstract

Freezing of gait (FOG) is one of the most disabling motor symptoms of Parkinson's disease, yet a fundamental question remains unresolved: why does FOG occur at any given moment? Most research has focused on studying FOG once it is already manifest. The present work aims to synthesize recent observations and propose a conceptual framework in which FOG reflects a dynamic process unfolding across multiple stages and time scales. Subtle changes in gait dynamics and physiology can precede episodes by several seconds (Stage −1), and experimental paradigms show that individuals without clinically recognized freezing may exhibit brief episodes under sufficiently sensitive conditions (Stage −2). At longer time scales, prospective studies indicate that vulnerability to freezing may be detectable years before onset (Stage −3). This framework reflects the idea that FOG emerges from progressive instability across interacting motor, cognitive, autonomic, and environmental systems, rather than as an isolated event. The proposed framework includes a prodromal stage, a subclinical stage, an immediate prefreezing state, and the freezing episode itself (Stage 0). This framework integrates findings from wearable sensing, physiology, and clinical studies and emphasizes that FOG remains latent in Stage −3, may be elicited only under specific conditions in Stage −2, and becomes manifest as instability evolves toward imminent and overt freezing (Stages −1 and 0, respectively). Reframing FOG as a multistage process that includes an upstream phase raises new questions regarding mechanisms, classification, and intervention, and may, ultimately, inform strategies aimed at predicting and preventing freezing rather than simply reacting to it.

## Graphical abstract

Freezing of gait (FOG) is conceptualized as an upstream process evolving from vulnerability (Stages −3, prodromal), to subclinical (Stage −2) to downstream FOG expression (Stages −1, pre‐FOG and Stage 0, FOG episode). Earlier stages reflect underlying susceptibility, whereas later stages reflect the dynamic unfolding of freezing episodes.

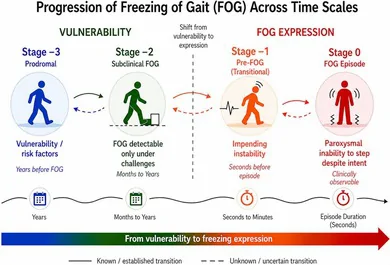

**Abbreviations**
AI: artificial intelligence
DBS: deep brain stimulation
EEG: electroencephalography
EMG: electromyography
FOG: freezing of gait
ICFOG: International Conference on Freezing of Gait
MCI: mild cognitive impairment
ON/OFF: medication states
PD: Parkinson's disease

## 1 Introduction

Freezing of gait (FOG) is one of the most disabling and poorly understood motor symptoms of Parkinson's disease (PD), contributing substantially to falls, reduced mobility, and loss of independence (Walton et al. 2015; Giladi et al. 2001; Nutt et al. 2011; Nieuwboer and Giladi 2013). The clinical definition of FOG has recently been revised by an expert panel as: “Paroxysmal episodes where there is an inability to step effectively, despite attempting to do so.” (Gilat et al. 2026). Furthermore, the same panel reached consensus on the need to characterize the heterogeneity of FOG and to enhance its conceptual clarity (Gilat et al. 2026). These efforts acknowledge that freezing is not a single, uniform phenomenon, but rather a context‐dependent breakdown in locomotor control that varies across individuals, tasks, and disease stages. However, despite decades of investigation, fundamental questions remain regarding its emergence, mechanisms, and temporal dynamics.

A central unresolved issue is why FOG emerges at one particular moment rather than another, despite the apparent availability of the motor, cognitive, and sensory capacities required to initiate or maintain gait. Clinically, episodes are often described as sudden and unpredictable, even in otherwise stable environments. However, this apparent abruptness may reflect limitations in how FOG has been studied and conceptualized. Much of the literature has focused on detecting FOG once it is already manifest or on characterizing its behavior during the episode itself (Sigcha et al. 2020; Bikias et al. 2021). While these approaches have yielded important insights, they implicitly frame FOG as a discrete event that begins when stepping visibly fails (Gilat et al. 2026). In contrast, converging evidence suggests that FOG may emerge from processes that evolve over time. Changes in gait dynamics and physiology can precede freezing by seconds (Handojoseno et al. 2013; Mazilu et al. 2013; Mazilu et al. 2015; Palmerini et al. 2017; Georgiades et al. 2019; Cao et al. 2021; Pardoel et al. 2024), while experimental paradigms demonstrate that individuals without self‐recognized FOG in daily life may exhibit brief episodes under sufficiently challenging experimental conditions (Mancini et al. 2023; Goris et al. 2025). At even longer time scales, longitudinal studies show that risk factors for FOG can be identified years before its clinical onset (Herman et al. 2023).

This perspective aligns with a broader distinction, long emphasized in public health, between downstream responses to manifest problems and upstream approaches that target underlying causes and earlier risk states (Rose 1985; McMahon et al. 2022; van Schalkwyk et al. 2026). The upstream–downstream framing is not new, but it remains useful because it captures a fundamental shift: from reacting to overt events toward identifying and modifying the processes that produce them. Applied to FOG, current efforts have largely focused on detecting and managing freezing episodes once they occur. While essential, this downstream focus may overlook or inadvertently de‐emphasize earlier stages in which vulnerability develops and transitions unfold. An upstream perspective instead emphasizes identifying and modifying the conditions that give rise to freezing, including prodromal vulnerability, subclinical manifestations, and the immediate transition states that precede overt episodes.

In the proposed framework, we refer to these stages as a temporal hierarchy, ranging from prodromal FOG (Stage −3), to subclinical FOG (Stage −2), to the pre‐FOG state (Stage −1), and finally to the overt freezing episode (Stage 0, the FOG state) (see Table 1 for definitions). To make this framework empirically testable while avoiding premature diagnostic cut‐points, we use observable features as operational anchors (Table 2). Stage −3 reflects heightened vulnerability to future FOG without objectively elicitable freezing; Stage −2 is marked by objectively observed freezing under sufficiently sensitive or provocative conditions despite the perceived absence of FOG in daily life; Stage −1 is the transient period immediately preceding an episode, when gait and/or physiological instability evolves toward FOG; and Stage 0 begins when ineffective stepping becomes overt and meets the contemporary clinical definition of a FOG episode. These anchors are intended to be testable rather than to imply discrete boundaries, and they may overlap or fluctuate across contexts and individuals.

**TABLE 1:** Conceptual stages of freezing of gait across temporal progression, including defining features, diagnostic visibility, and stability characteristics.

Stage	Approximate time scale	Phenomenology	Diagnostic visibility	Stability/modulation
Stage −3 (Prodromal)	Years	Early risk factors and emerging vulnerability to freezing, including motor, cognitive, affective, and other nonmotor changes	No objectively elicitable freezing, including under appropriately provocative conditions	Relatively stable, with gradual progression over time
Stage −2 (Subclinical)	Months or years	Brief or subtle objectively adjudicated freezing elicited under sensitive or provocative conditions, without recognized FOG in daily life	FOG not recognized in daily life or routine examination; becomes detectable under high challenge	Highly context‐dependent; fluctuates with cognitive load, arousal state, environmental demands, and medication state (ON/OFF)
Stage −1 (Pre‐FOG)	Seconds to minutes	Immediate pre‐episode instability, including gait degradation and physiological changes preceding freezing	Detectable immediately before FOG using real‐time or continuous monitoring	Transient and rapidly evolving; reflects dynamic instability
Stage 0 (FOG)	Momentary	Overt freezing episode, including motor arrest or trembling of the legs (see Gilat et al. 2026)	Clinically observable under routine, provocative, or naturalistic conditions; may be perceived/reported by the patient	Episodic; may resolve spontaneously or in response to interventions, e.g., cues

**TABLE 2:** Candidate measures and operational anchors for the proposed FOG stages. Measures listed as candidates are not necessarily validated diagnostic biomarkers or individual‐level predictors; longitudinal validation is especially needed for Stage −3. Representative supporting references for each stage are provided below the table.

Stage	Proposed operational anchor	Candidate measures/approaches	Illustrative heterogeneity and interpretation
Stage −3 (Prodromal)	Heightened future FOG vulnerability, but no objectively elicitable freezing even under appropriately sensitive or provocative testing.	Longitudinal clinical and motor risk factors; repetitive motor‐control and quantitative gait/turning measures; executive‐attentional and other nonmotor measures; digital mobility monitoring; structural MRI (including thalamic morphology); and candidate functional‐connectivity and EEG measures. These are not yet validated for individual‐level prediction.	Potential motor‐dominant, cognitive‐executive, affective/autonomic, or context‐sensitive vulnerability profiles. Many candidate markers are nonspecific to FOG and must ultimately predict progression to Stage −2 or Stage 0 beyond general PD severity and other causes of gait impairment.
Stage −2 (Subclinical)	Objectively adjudicated FOG can be elicited under sufficiently challenging conditions despite the absence of recognized FOG in daily life or routine examination.	Provocative paradigms (e.g., challenging 360° turning, dual‐tasking, box shuffle/agility, doorway negotiation); synchronized video or expert clinical adjudication; sensitive wearable kinematics and related digital measures; comparison of self‐report with objective observation.	May represent an early progressive state, a fluctuating context/medication‐dependent state, or a relatively stable high‐resilience phenotype. Detection is strongly dependent on task design, definitions, and adjudication.
Stage −1 (Pre‐FOG)	Transient seconds‐to‐minutes period immediately preceding an episode, when compensatory control begins to fail and FOG becomes imminent.	Short‐term changes in stride variability, bilateral coordination and turning dynamics; wearable‐sensor and machine‐learning prediction; heart rate/autonomic measures; EEG; EMG; subthalamic local‐field potentials and cortico‐subthalamic coherence.	Gradual versus relatively abrupt transitions; trigger‐specific patterns (turning, gait initiation, cognitive load); within‐person variability in lead time and physiological signatures.
Stage 0 (FOG)	Overt ineffective stepping consistent with the contemporary clinical definition of a FOG episode.	Clinical/video adjudication; episode frequency, duration and percent time frozen; triggers and manifestations; wearable/algorithmic detection; concurrent EEG, EMG, autonomic or local‐field‐potential measures when available.	Akinetic, kinetic‐trembling, and kinetic‐no‐trembling manifestations; variable triggers, duration, medication responsiveness, recovery mechanisms, and clinical impact.

*Note:* Representative supporting references (not exhaustive): Stage −3: D'Cruz et al. (2020); D'Cruz et al. (2021); Herman et al. (2023); Mancini, Afshari, et al. (2025). Stage −2: Mancini et al. (2023); Goris et al. (2025); Howe et al. (2026), in press. Stage −1: Mazilu et al. (2015); Palmerini et al. (2017); Economou et al. (2021); Moore et al. (2024); Pardoel et al. (2024); Mancini, McKay, et al. (2025). Stage 0: Salomon et al. (2024); Mancini et al. (2025); Gilat et al. (2026).

The stages themselves are also unlikely to be homogeneous. Instead, they include heterogeneous individual profiles within each stage, and the trajectories linking stages may also differ across individuals. The proposed model is summarized in Table 1 and illustrated conceptually in Figure 1. This article provides a focused narrative synthesis rather than an exhaustive review of the FOG literature. In the following sections, we synthesize the evidence supporting this framework, highlight key open questions, and discuss its implications for measurement, mechanisms, and intervention. Importantly, the aim of this work is not to address prevention strategies per se, but rather to provide a conceptual framework for staging and characterizing earlier phases of FOG across time scales.

**FIGURE 1:**
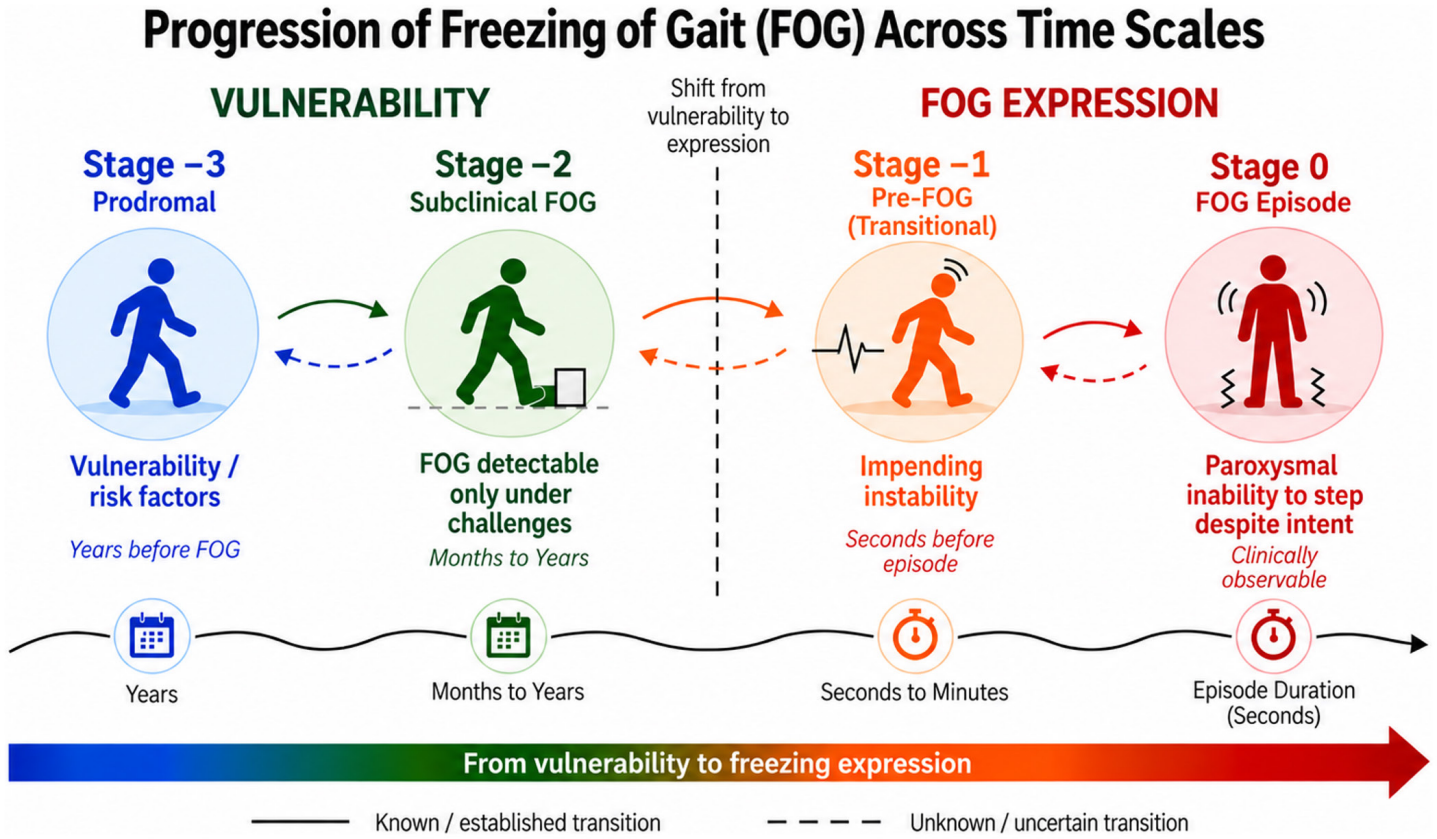
A multistage framework of freezing of gait (FOG) across time scales. FOG is conceptualized as a process evolving from vulnerability (Stages −3 and −2) to FOG expression (Stages −1 and 0). Earlier stages reflect underlying susceptibility, whereas later stages reflect the dynamic unfolding of freezing episodes. Stage −3 (prodromal) represents vulnerability and risk factors in the absence of detectable freezing, while Stage −2 (subclinical FOG) reflects a latent state in which freezing may be elicited under sufficiently sensitive or provocative conditions. Stage −1 represents a transitional phase immediately preceding a freezing episode, characterized by impending instability, and is best understood as part of the freezing cascade rather than a stable disease stage. Stage 0 corresponds to overt freezing episodes, defined as a paroxysmal inability to step effectively despite the intention to walk. Importantly, progression is likely not strictly linear: individuals may fluctuate between stages, and features of later stages (including pre‐FOG or overt freezing) may emerge within earlier stages under specific task, environmental, or physiological conditions, particularly in Stage −2. Transitions may also occur in both directions, including returns from Stage 0 to Stage −1, consistent with observations of repeated or clustered episodes interspersed with transient pre‐FOG states. The mechanisms governing the termination of a freezing episode remain incompletely understood, and in some cases, successive events may form a cascade, raising unresolved questions about when one episode ends and another begins. This framework emphasizes that FOG may remain latent and context‐dependent, emerging only under sufficiently challenging circumstances. Although individuals with Parkinson's disease are generally at increased risk of developing FOG, Stage −3 refers more specifically to a subset with heightened vulnerability relative to other patients, highlighting the potential for early identification and stratification.

## 2 The Freezing Episode (Stage 0)

Much of the earlier work focused on identifying differences between individuals with and without freezing, what occurs during a FOG episode, and the consequences of FOG (Giladi et al. 2000; Schaafsma et al. 2003). More recent advances in sensing and experimental paradigms have enabled increasingly detailed study of the freezing episode itself, the most clinically visible manifestation of this disabling symptom. Episodes are typically brief yet highly disruptive, often involving a complete movement cessation, nowadays referred to as the “akinetic *core*” (Gilat et al. 2026). However, FOG episodes may also manifest as transient periods of high‐frequency tremulous oscillatory movements (“kinetic‐trembling *FOG*”) or ineffective shuffling steps without trembling (“kinetic‐no‐trembling *FOG*”), which may occur with or without an accompanying core (Nutt et al. 2011; Snijders et al. 2012; Gilat et al. 2026). In practice, FOG may occur when a person initiates walking, turns, approaches a doorway or narrow passage, or walks under increased cognitive demand. A typical episode lasts only a few seconds, although longer events occur, and effective forward stepping becomes markedly reduced or absent despite the intention to continue walking. The episode may appear as an akinetic arrest, trembling of the legs, or ineffective shuffling steps, providing a concrete clinical reference for the subtler changes considered in the pre‐FOG state as indicated below.

Experimental paradigms consistently demonstrate that freezing is most likely to occur when the motor system is under high demand or in conditions that challenge the integration of motor, cognitive, and environmental input, such as turning, dual‐task walking, or navigating constrained spaces (Shine et al. 2013; Lewis and Shine 2016; Conde et al. 2024; Zoetewei, Herman, et al. 2024). Furthermore, FOG is more likely to emerge when the individual's emotional state is under heightened arousal or stress (Ehgoetz Martens et al. 2020). These findings support the view that freezing reflects a paroxysmal failure of the locomotor network and its interactions with attentional, sensory, and limbic systems, rather than an isolated functional motor deficit.

Substantial progress has been made in the objective detection of freezing episodes through wearable sensors and machine‐learning approaches (Mancini, McKay, et al. 2025). Inertial measurement units enable continuous monitoring, and increasingly sophisticated algorithms can identify freezing based on characteristic movement patterns such as high‐frequency oscillations, reduced stride amplitude, and altered coordination (Sigcha et al. 2020; Bikias et al. 2021). Reviews and benchmark studies make clear that detection has improved, but they also show that performance is highly dependent on sensor placement, task context, class imbalance, and reference labels (O'Hanlon et al. 2024). These constraints become more pronounced in daily living contexts, where variability is greater, and apparently similar events may differ in their clinical consequences (Adams et al. 2021; Morris et al. 2012; Denk et al. 2022).

Despite these advances, several fundamental questions remain unresolved (Tables 3A and B). Among the most important open questions are the precise neural and biomechanical processes underlying the onset and termination of the core of a freezing episode (Lewis and Shine 2016). It also remains unclear why similar perturbations lead to freezing in some instances but not in others, even within the same individual (Zoetewei, Ginis, et al. 2024). Finally, the mechanisms that enable recovery from freezing, often occurring abruptly, are poorly characterized. These unresolved issues suggest that focusing on the episodes is informative but also limits progress and motivates a broader perspective that includes the processes leading into and out of freezing. In this context, the freezing episode can be viewed not as an isolated event, but as the final manifestation of processes unfolding over multiple time scales.

**TABLE 3A:** Selected open questions within the framework stages.

Domain	Key questions	Example approaches
Understanding risk (Stage −3)	Which early markers predict the development of FOG, and how early do these changes emerge? Can the risk be prevented?	Longitudinal cohort studies with external validation; cognitive, affective, and nonmotor assessments; digital mobility measures
Subclinical detection (Stage −2)	How can subclinical FOG be reliably identified? What distinguishes subclinical freezing from mild or context‐dependent clinical FOG? Is this stage modifiable? How long does this period last?	Provocative paradigms (e.g., turning, dual‐tasking, and box shuffle); wearable sensors; synchronized video validation
Pre‐FOG prediction (Stage −1)	Which signals best predict imminent freezing? Are there reproducible variants of pre‐FOG dynamics?	Wearable sensing; machine learning approaches; multimodal physiological monitoring (e.g., EEG, EMG, and autonomic signals)
Overt FOG (Stage 0)	What allows the person to end the FOG episode? What impacts its duration, manifestations and core? How do FOG severity and manifestations evolve over time?	Wearable sensing; machine learning approaches; multimodal physiological monitoring (e.g., EEG, EMG, and autonomic signals). Real‐time measurement with EEG and local field potentials

## 3 The Pre‐Fog State (Stage −1)

A growing body of evidence supports the existence of a pre‐FOG state, characterized by measurable changes in motor and physiological signals in the seconds preceding a freezing episode. Early studies demonstrated progressive gait degradation prior to freezing, including increased stride variability, reduced bilateral coordination, and altered turning dynamics (Mazilu et al. 2013; Palmerini et al. 2017). More recent work has extended these findings using wearable sensors and machine‐learning approaches, showing that freezing can be predicted several seconds before onset, typically within a 1–5 s window (Mazilu et al. 2015; Shalin et al. 2021; Pardoel et al. 2024). These findings suggest that freezing is often preceded by detectable changes, rather than occurring entirely without warning. In other words, Stage −1 can be conceptualized as the immediate pre‐freezing state, reflecting a transitional phase within the broader freezing cascade rather than as a discrete autonomous stage. The revised onset definition of FOG, which characterizes the episode as the first failure to initiate a step or the emergence of clearly ineffective stepping, further emphasizes that the exact demarcation between pre‐freezing and freezing is increasingly indistinct (Gilat et al. 2026).

The pre‐FOG literature also reveals substantial heterogeneity in this critical phase. In some individuals, freezing is preceded by gradual and consistent changes in gait, whereas in others the transition appears more abrupt or less predictable. This variability raises the possibility that multiple pathways to freezing exist, potentially reflecting differences in manifestations, triggers, disease stage, medication state, and underlying neural mechanisms (Shine et al. 2013; Lewis and Shine 2016). In addition, there are task‐related pre‐FOG states that require a different kind of motor control. For example, turning‐related freezing may involve different cycle‐to‐cycle gait dynamics than freezing during gait initiation or under cognitive load (D'Cruz et al. 2022; Zoetewei, Ginis, et al. 2024). Subtype‐oriented work is consistent with this broader idea of heterogeneity and suggests that different combinations of motor and freezing‐related features may define partially distinct phenotypes (Ehgoetz Martens, Shine, et al. 2018; Zoetewei et al. 2025). This possibility is important because it implies that a single pre‐FOG model, or a uniform lead time, may be unrealistic. It is also conceivable that distinct pre‐freezing states may converge onto a common pathway through which FOG is either triggered or successfully resisted (Lewis and Shine 2016).

Physiological studies further support a multi‐system view of the pre‐FOG state. Changes in autonomic activity, cortical dynamics, cortico‐subthalamic coherence, and muscle activation patterns have been observed before freezing, indicating involvement of multiple interacting systems (Maidan et al. 2010; Economou et al. 2021; Handojoseno et al. 2013; Moore et al. 2024). Heart‐rate studies suggest that at least some episodes are preceded by anxiety‐linked autonomic arousal (Maidan et al. 2010; Economou et al. 2021; Cockx et al. 2023). Furthermore, local field potentials registered in the subthalamic nucleus, electroencephalography (EEG), and electromyography (EMG) studies suggest that impaired inhibitory control, altered supraspinal contributions to locomotor regulation, and abnormal cortical, subthalamic, and muscular dynamics may all precede overt freezing (Georgiades et al. 2023; Klocke et al. 2024). These observations move the field beyond a purely gait‐based account and support the idea that Stage −1 is a cumulative neurobehavioral state transition rather than merely a kinematic threshold crossing.

These findings are consistent with a dynamic network perspective in which freezing reflects a transient breakdown in coordination across neural systems. Prior work suggests that freezing is associated with functional decoupling between cognitive control networks and basal ganglia circuits, reflecting impaired integration within the locomotor control systems (Shine et al. 2013; Lewis and Shine 2016). Taken together, the pre‐FOG state may reflect increasing instability or ‘noise’ in these interactions, reducing the system's ability to resolve competing motor programs and decreasing resilience to perturbations.

From this perspective, the immediate instability observed in the pre‐FOG state may reflect inadequate ‘neural reserve’ (Hoenig et al. 2023), or a failure to leverage compensatory mechanisms that had been fit for purpose in masking underlying vulnerability at earlier stages (Factor et al. 2025; Tosserams et al. 2025). More specifically, freezing may emerge when cognitive, motor, sensory, and limbic demands exceed available control resources, consistent with interference or overload models of gait regulation (Ehgoetz Martens, Hall, et al. 2018; Taylor et al. 2022). This shift suggests that Stage −1 represents a tipping point at which compensatory control breaks down, allowing latent instability to manifest as overt freezing.

This interpretation is also consistent with the classic threshold‐based models of freezing in which accumulating gait abnormalities, such as increased variability and impaired coordination, push locomotion toward a point of instability beyond which freezing occurs (Plotnik et al. 2012). Recent work in the context of adaptive deep brain stimulation (DBS) also provides indirect support for this model, demonstrating that kinematically triggered increases in stimulation during periods of gait irregularity have the potential to reduce gait disruption (Fekri Azgomi et al. 2025). These findings suggest that dynamic modulation of neural circuits near this instability threshold may alter the propensity of transitioning into manifest freezing, reinforcing the idea that Stage −1 reflects a critical juncture in system dynamics that can be targeted with future on‐demand interventions (Zoetewei, Herman, et al. 2024).

Context is also likely to play an important role in this pre‐FOG stage. Environmental constraints, dual‐task demands, emotional state, time of day, time since last medication dose, fatigue, and sleep‐related factors may all modulate the probability that ongoing instability progresses to observable freezing (Conde et al. 2024; Monaghan et al. 2023; Zitser et al. 2025). It is plausible that contextual influences differ across individuals and even within the same individual across days or situations. This observation may help explain why FOG detection in daily‐living settings differs from detection during structured laboratory‐based paradigms (Mancini et al. 2021). More generally, it suggests that the pre‐FOG state cannot be defined solely by a characteristic signal pattern but rather emerges from the interaction between that pattern and the contextual demands placed on the locomotor system.

As summarized in Table 3A, several open questions follow directly from the above reasoning. Are there reproducible variants of pre‐FOG signals? Do autonomic or cortical signatures map onto specific behavioral triggers? How stable are pre‐FOG patterns within individuals over time? Perhaps, most importantly, can pre‐FOG detection be translated into an actionable intervention to prevent or reduce the severity of an impending FOG episode? Can adaptive deep brain stimulation stabilize brain oscillations in the pre‐freezing state to prevent progression to evident freezing (Klocke et al. 2025) (Table 3B)? Answering these questions will be critical for moving from proof‐of‐concept predictions toward clinically meaningful prevention strategies that are tailored to individual profiles.

**TABLE 3B:** Selected open questions across the staging framework.

Domain	Key questions	Example approaches
Pathophysiology (across stages)	How do motor, cognitive, and autonomic systems interact across stages, and what mechanisms drive transitions between them?	Experimental paradigms; network‐based modeling; integration of kinematic and physiological data
Neurobiological signatures	What longitudinal changes in neural systems (e.g., cholinergic, GABAergic, noradrenergic, frontostriatal, and brainstem networks) track progression across stages −3 to 0?	PET imaging; EEG/fNIRS; local field potentials; multimodal longitudinal designs
Trajectories and heterogeneity	Are stage transitions linear, fluctuating, or multidirectional, and do distinct variant profiles follow different trajectories?	Longitudinal modeling; clustering and subtype analyses; individualized trajectory mapping
Within‐person variability and state dependence	Why do similar perturbations lead to freezing in some instances but not others, even within the same individual?	Multimodal longitudinal studies incorporating context and continuous monitoring
Brain‐sensing	Which multimodal biomarkers would allow adaptive deep brain stimulation to target the pre‐freezing state in order to prevent overt freezing?	Stabilizing brain oscillations with adaptive deep brain stimulation
Clinical translation	Can early‐stage detection inform intervention, and what endpoints are appropriate for trials targeting earlier stages?	Integration with clinician‐ and patient‐reported outcomes; real‐world monitoring; intervention studies

Abbreviations: EEG = electroencephalography; EMG = electromyography; fNIRS = functional near‐infrared spectroscopy; FOG = freezing of gait; PET = positron emission tomography.

## 4 Subclinical Freezing of Gait (Stage −2)

Beyond the immediate pre‐FOG state, evidence suggests that freezing may be present before it is clinically recognized or perceived by patients themselves, supporting the concept of subclinical FOG (Stage −2). At this stage, and under sufficiently sensitive or provocative conditions, elements of the pre‐freezing stage (Stage −1) and even of overt freezing (Stage 0) may be observed, reflecting the dynamic and context‐dependent nature of the transition between states. Consistent with this concept, evidence indicates that individuals classified as nonfreezers may, nevertheless, exhibit brief freezing episodes, particularly during full‐body turning or highly cognitively demanding tasks (Mancini et al. 2023; Goris et al. 2025). These observations challenge the traditional binary classification of patients and instead support a continuum of freezing susceptibility, in which clinically apparent freezing represents only the most visible portion of a broader underlying process. In this framework, subclinical FOG is defined by the absence of recognized FOG during daily life and limited detectability in routine clinical practice, while objectively adjudicated FOG becomes evident when the gait task or context is sufficiently challenging. This objectively elicitable freezing provides the proposed operational anchor separating Stage −2 from Stage −3; in the latter, vulnerability may be measurable, but freezing itself cannot yet be elicited.

Pharmacological factors may further contribute to the emergence and detection of subclinical FOG. Levodopa and other dopaminergic therapies may suppress or mask freezing in some individuals (Tosserams et al. 2025; Gilat et al. 2026), potentially contributing to a “grey zone” in which vulnerability is present but not consistently expressed under routine conditions (Plotnik et al. 2012). This raises the possibility that subclinical FOG may, in part, reflect a state‐dependent expression of freezing susceptibility, which is dependent upon the neural reserve across networks and modulated by dopamine availability and its interaction with cognitive and environmental demands.

This framework also helps to explain findings in which individuals revert from clinically apparent freezing to a subclinical state over time. Indeed, a study by Hulzinga et al. (2020) demonstrated that seven of 57 participants transitioned from being initially self‐reported freezers to nonfreezers within a 6‐week period, for reasons that remained unclear. In a similar vein, 33 out of 55 patients with self‐reported FOG reverted to a subclinical nonfreezing state following DBS over a 24‐month period (Barbe et al. 2020). A comparable conversion was also noted in 15 out of 56 self‐reported freezers following an increase in levodopa dosage (Barbe et al. 2020). Somewhat paradoxically, in some patients with PD, levodopa may lead to or trigger FOG (Jansen et al. 2023), underscoring the heterogeneity of this symptom's mechanisms.

Additional evidence for a continuum of freezing susceptibility rather than a rigid dichotomy was provided by Mancini and colleagues, who showed that digital mobility measures capturing gait breakdown span a graded spectrum from nonfreezers to possible and definite freezers (Mancini et al. 2023). Notably, while some gait and turning impairments were similar across these groups, the Freezing Index, a metric that reflects the relative dominance of high‐frequency leg movement over normal locomotor frequencies, did distinguish between these groups and healthy controls. This finding suggests that broader locomotor impairments may reflect upstream vulnerability to freezing and raises important questions about the specificity and validity of digital markers to detect subclinical FOG. In other words, the vulnerability to develop freezing may be distributed across multiple mobility domains rather than residing in a single metric. Importantly, the same quantitative gait and turning measures may also be informative in Stage −3, but their interpretation differs: in the absence of elicitable FOG, they are candidate markers of future vulnerability rather than evidence of subclinical freezing. Prospective follow‐up is therefore needed to determine whether such measures predict transition from Stage −3 to Stage −2 or Stage 0.

Complementary findings from Goris and colleagues further reinforce this interpretation. Using a cognitively demanding 360° turning paradigm for 1 min in both directions, they showed that 63.1% of self‐reported nonfreezers exhibited expert‐rated freezing, while having no subjective awareness of the symptom (Goris et al. 2025). Interestingly, the above‐mentioned Freezing Index had limited classification performance and only weak criterion validity against video‐rated time spent freezing (Goris et al. 2025). These findings are highly informative for several reasons: challenging turning tasks may be effective for revealing latent or early freezing, but some of the metrics commonly used to quantify freezing‐related phenomena remain insufficiently robust to serve as a stand‐alone ground truth to capture freezing. This limitation matters for both research design and interpretation and is consistent with ongoing efforts to improve automated detection and clinician‐ and patient‐reported outcome measures for FOG (Giladi et al. 2009; Nieuwboer et al. 2009; Mancini, McKay, et al. 2025; Salomon et al. 2024; Ehgoetz Martens et al., n.d.‐a, n.d.‐b).

As illustrated in Figure 2, our exploratory findings based on unpublished interim data from the ongoing FOG‐COA study are roughly consistent with Goris's findings. In interim analyses using the eight‐task GP‐FOG provoking protocol described by Howe et al. (2026, in press), 10 of 42 participants classified as nonfreezers by self‐report exhibited a small number of freezing episodes based on an on‐the‐spot (real‐time) clinical assessment of FOG. Although these observations should be interpreted cautiously until final adjudication is complete, they align directionally with the previous findings (Goris et al. 2025), suggesting that clinically unrecognized or unreported freezing may only become detectable under sufficiently provocative conditions, supporting the presence of a subclinical state. However, the proportion of protocol‐elicited freezing varies substantially across studies, approximately one‐quarter in our interim data compared with 63.1% in Goris et al., a discrepancy that likely reflects differences in task design, FOG definition, detection methodologies, and cohort characteristics. Together, these observations highlight the sensitivity of subclinical FOG detection to patients' self‐perception, experimental paradigms, operational definitions, and adjudication criteria.

**FIGURE 2:**
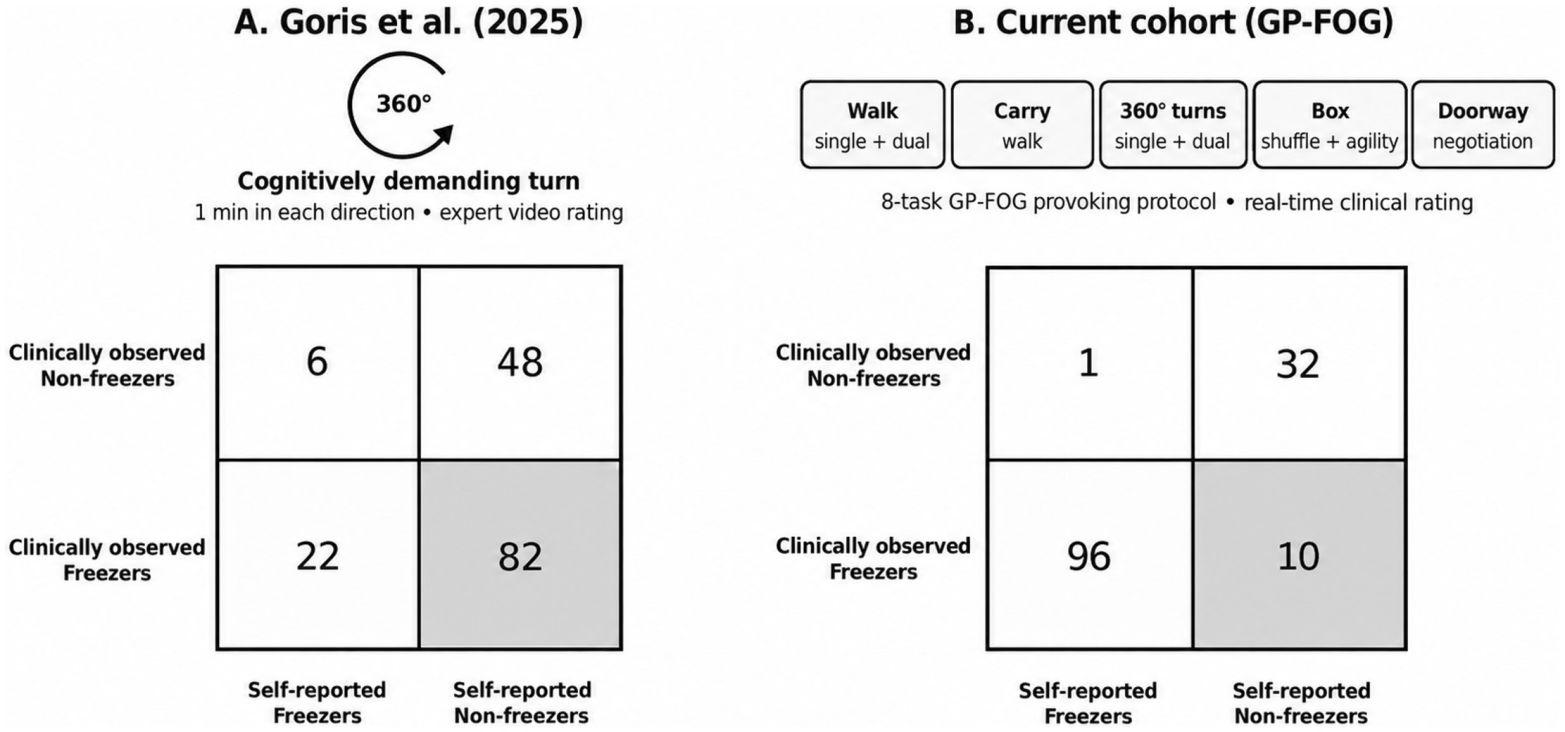
Discordance between clinical classification and freezing of gait in two different studies. (A) In an independent cohort (Goris et al. 2025), FOG was elicited using a cognitively demanding 360° turning paradigm performed for 1 min in each direction and identified by expert video rating. (B) In the present cohort from the ongoing FOG‐COA study, participants completed the eight‐task GP‐FOG provoking protocol described by Howe et al. (2026, in press): single‐task walk, dual‐task walk, carrying walk, alternating 360° turns, dual‐task alternating 360° turns, box shuffle, box agility, and doorway negotiation; FOG was identified by an on‐the‐spot clinical assessment. FOG was clinically observed in 104 individuals in the Goris cohort, including 82 who did not report freezing, and was elicited in 10 of 42 individuals classified as nonfreezers by self‐report in the present cohort. Taken together, 63.1% (A) and 23.8% (B) of self‐reported nonfreezers unexpectedly exhibited FOG, illustrating that FOG may remain latent and become apparent only under sufficiently challenging conditions. The differing task designs and adjudication methods are important and preclude a direct quantitative comparison of the two proportions. The Figure 2B findings are unpublished interim data from a multisite study approved by the respective institutional review boards at each participating site and conducted in accordance with the Declaration of Helsinki.

The existence of subclinical FOG also raises important questions regarding trajectories across stages. One possibility is that subclinical FOG represents an early phase that precedes clinically apparent freezing. It might also reflect a fluctuating state in which individuals move in and out of freezing susceptibility depending on context, medication state, or cognitive load. A third possibility is that subclinical FOG represents a relatively stable phenotype in some individuals, distinct from those who progress to overt freezing, due to enhanced and sustained neural resilience in these subjects. These possibilities are not mutually exclusive, and they suggest that both stage and subtype may be necessary to fully characterize freezing vulnerability. Longitudinal work will be required to determine how often Stage −2 progresses to Stage 0 or vice versa, how often it fluctuates, and which markers best predict these transitions.

An additional implication concerns the interpretation of false positives in automated FOG‐detection, especially in real‐world monitoring studies. In continuous mobility data, a proportion of detected events are typically labeled as false positives (Mazilu et al. 2015; Mancini et al. 2023; Pardoel et al. 2024; Salomon et al. 2024). However, some of these events may not reflect purely algorithmic error but instead capture subtle or context‐dependent manifestations of freezing that fall below the threshold of conventional clinical recognition. As such, they may represent subclinical or transitional freezing states rather than true misclassifications. This interpretation does not eliminate the problem of false positives, but it suggests that further investigation is warranted using validated gold standard measures of FOG to better delineate the boundary between algorithmic error and early biological signal in naturalistic studies. Recent systematic evaluations of digital health technologies further highlight both the promise and limitations of current approaches for measuring parkinsonian motor symptoms, including FOG (Virameteekul et al. 2026).

Together, these findings suggest that vulnerability to freezing may already be present even when it is not consistently expressed (Mancini et al. 2023; Goris et al. 2025). Future analyses will examine whether these individuals exhibit elevated levels of known risk factors already present in earlier stages, such as cognitive impairment, mood symptoms, or sleep disturbances, and whether, and over what time course, they transition to clinically apparent FOG in daily life.

## 5 Prodromal FOG (Stage −3)

At longer time scales, freezing likely emerges from processes developing over years before its clinical manifestation, consistent with a prodromal stage (Stage −3). Longitudinal studies have already identified predictors of incident FOG, including motor severity, disease duration, cognitive impairment, depression, anxiety, levodopa dose, and neuroimaging markers (Herman et al. 2023; Amboni et al. 2015; Ehgoetz Martens, Lukasik, et al. 2018; D'Cruz et al. 2020; D'Cruz et al. 2021; Monaghan et al. 2023; Faerman et al. 2025). These findings indicate that vulnerability to freezing can be detected, at least on a group level, well before overt symptoms appear and suggest that freezing may reflect progressive changes across multiple domains rather than a sudden conversion from normal to abnormal gait. Remarkably, some changes have been seen 12 years before FOG manifests (Herman et al. 2023). Longitudinally supported candidate measures include repetitive motor‐control and gait measures, cognitive and affective features, and structural neuroimaging measures such as thalamic morphology (D'Cruz et al. 2020; D'Cruz et al. 2021; Herman et al. 2023). Recent work on digital gait biomarkers also highlights their potential for characterizing susceptibility/risk and prognosis in PD (Mancini, Afshari, et al. 2025); however, FOG‐specific individual‐level predictive validity remains to be established. Functional connectivity abnormalities in gait‐related networks have also been described in established FOG (Sreenivasan et al. 2023), but whether these or EEG‐based measures can improve such prediction also remains uncertain.

FOG has been associated with dysfunction in multiple neural systems, including frontostriatal circuits, brainstem locomotor regions, and cholinergic pathways (Nutt et al. 2011; Lewis and Shine 2016; Bohnen et al. 2019; Bardakan et al. 2022). These systems are critical for integrating motor, cognitive, and sensory inputs during walking. Emerging evidence also highlights the contribution of nonmotor factors, including cognition and sleep disturbances, to freezing vulnerability (Monaghan et al. 2023; Zitser et al. 2025). The recent literature on cognition and FOG is especially relevant here, as it suggests that executive‐attentional dysfunction is not simply a correlate of established freezing, but may be part of the broader substrate that predisposes to it (Craig et al. 2026; Wang et al. 2026). Similarly, discussion of sleep disturbance and FOG invites the possibility that vulnerability may be shaped by processes that unfold well outside the moments in which freezing is observed (Zitser et al. 2025).

The role of context, nonmotor symptoms, and broader biology may be especially important in Stage −3. Environmental exposures, stress, fatigue (Faerman et al. 2025), medication variability, and fluctuating symptom burden (Loo et al. 2025) may all interact with a predisposing substrate (Ehgoetz Martens, Lukasik, et al. 2018; Herman et al. 2023). In addition, the potential roles of genetics and epigenetics remain largely unexplored. While current evidence is insufficient to define specific pathways (Factor et al. 2011; Radojević et al. 2022), these genetic factors may contribute to inter‐individual variability in freezing risk, phenotype, and progression. Integrating such factors into future longitudinal studies will be important for understanding individual variability.

An important challenge is specificity. Many candidate Stage −3 features, including slower gait, greater gait variability, and executive‐attentional impairment, are not unique to FOG and may accompany general PD progression, aging, cognitive impairment, or other neurological gait disorders. For example, gait abnormalities during usual, dual‐task, and destabilizing walking have also been described in older adults with higher‐level gait disorders and cognitive impairment (Zhang et al. 2025). Thus, an isolated gait, cognitive, MRI, or physiological abnormality should not presently be interpreted as prodromal FOG. A useful Stage −3 marker or index based on multiple markers will ultimately need to predict subsequent subclinical or overt FOG beyond general disease severity and competing causes of gait impairment, likely requiring multimodal longitudinal models.

Apart from prediction studies, the concept of prodromal FOG is still largely theoretical and raises several important questions (Herman et al. 2023). What are the earliest detectable markers of freezing vulnerability? How specific are they to freezing as opposed to general disease progression? Alternatively, in the absence of a single marker that does not also reflect disease severity, can a synthesized index be generated to identify people with the greatest risk of developing FOG in the future? What prodromal factors are modifiable (Tosserams et al. 2026)? In line with what is discussed above, it is highly plausible that prodromal FOG is heterogeneous, with different pathways leading to freezing. Some individuals may develop freezing primarily through motor network dysfunction, whereas others may be more strongly influenced by cognitive, autonomic, affective, or contextual factors. Candidate approaches include executive‐attentional testing (e.g., interference control and set shifting), quantitative gait and turning measures, structural and functional neuroimaging, EEG and other physiological measures, and digital mobility monitoring; however, their predictive value is not equivalent and most require prospective validation for individual‐level prediction (Table 2). Addressing these questions will require longitudinal, multimodal studies that integrate digital mobility measures, clinical assessments, and physiological data, using updated definitions (Gilat et al. 2026) to quantify and classify FOG conversion as outlined conceptually in Tables 1, 2, 3A, and B.

The distinction between prodromal and subclinical FOG may be understood by analogy to mild cognitive impairment (MCI) in the context of Alzheimer's disease, although the analogy is not perfect. MCI is associated with an increased risk of progression to dementia, yet many individuals with MCI do not develop Alzheimer's dementia, and some even revert, possibly temporarily, to cognitively intact states. Similarly, subclinical FOG and, by extension, prodromal FOG, may reflect an increased vulnerability to freezing that does not inevitably progress to overt FOG in all patients. Transitions along this continuum are likely influenced by factors such as age, cognitive reserve, environmental demands, disease progression rates, physical fitness and activity, and other compensatory mechanisms. This perspective supports the notion that identifying and targeting individuals in these earlier stages may offer opportunities for intervention before irreversible clinical manifestations emerge. As with MCI, the development of risk prediction models could help to identify individuals most likely to develop clinical freezing, thereby enabling targeted early intervention. Predictive measures could generate an individualized risk score or assemble a prodromal FOG severity index based on dimensions known to contribute to FOG. Such measures would enable formal testing of the hypothesis that individuals who develop subclinical FOG within the ensuing months or years exhibit greater prodromal severity than those who do not manifest subclinical FOG. Importantly, estimating time to phenoconversion would allow evaluation of whether interventions not only modify the risk of progression but also alter the estimated time to transition.

## 6 Discussion

The staging model proposed here suggests that FOG is best understood as a process evolving from Stage −3 through Stage 0 (Figure 1). While each stage is supported by prior empirical findings, the proposed framework integrates these observations across time scales and generates testable hypotheses rather than proposing validated diagnostic categories or fixed cut‐points. This perspective introduces a conceptual shift that distinguishes underlying susceptibility from the dynamic evolution of freezing events. Earlier stages (−3 and −2) primarily reflect vulnerability and reduced neural reserve, whereas later stages (−1 and 0) capture the unfolding of a freezing episode under specific contextual demands. From this perspective, Stage −1 is best understood as a transitional phase within the freezing cascade, marking the point at which compensatory control begins to fail and a freezing episode becomes imminent. Importantly, subclinical FOG (Stage −2) will, in practice, include elements of Stages −1 and 0, when assessed under sufficiently sensitive conditions, consistent with a continuous spectrum characterized by partially overlapping mechanisms. Operationally, the emergence of objectively elicitable freezing distinguishes Stage −2 from Stage −3, whereas Stage −1 captures the moment‐to‐moment dynamics immediately preceding an actual freezing episode. The current framework therefore highlights the need to distinguish underlying vulnerability from elicitable subclinical expression and from the rapid dynamics that culminate in overt FOG.

The implications of this conceptual model are twofold. First, it shifts research attention from downstream manifestations of freezing to upstream processes, although doing so necessitates prospective follow‐up studies in large cohorts. Second, it reframes the treatment approach to align with renewed public‐health arguments that advocate targeting root causes at earlier disease states, rather than reacting once symptoms are fully manifest (Rose 1985; McMahon et al. 2022; van Schalkwyk et al. 2026). Still, even if much work to date has focused on Stage 0, it is encouraging to note that the most recent evidence‐based treatment recommendations for freezing management endorse elements of the reasoning articulated here, signaling emerging consensus within the field (Herman et al. 2023; Tosserams et al. 2025). The clinical impact, urgency, and fall risk associated with overt freezing episodes make them an essential treatment target. However, an exclusive focus on manifest freezing may unnecessarily narrow the therapeutic window. An upstream perspective instead emphasizes the identification of vulnerability and transition states before freezing becomes observable, thereby opening new opportunities for prediction, risk stratification, and early intervention, as is illustrated in Figure 3.

**FIGURE 3:**
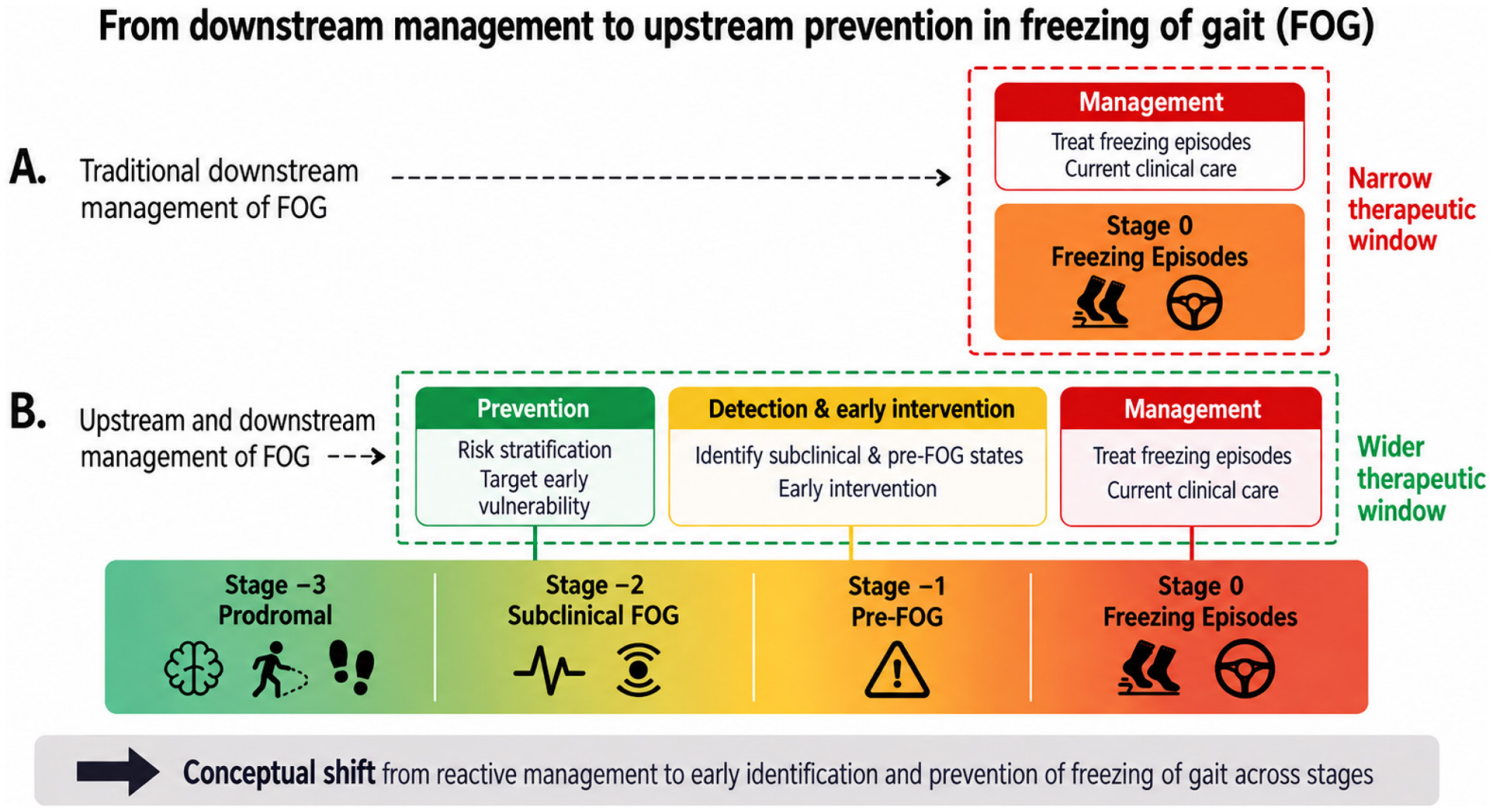
From downstream management to upstream prevention in freezing of gait (FOG). (A) Traditional approaches primarily focus on managing overt freezing episodes (Stage 0), representing a relatively narrow therapeutic window. (B) A stage‐based framework highlights opportunities for intervention across earlier stages, including prodromal vulnerability (Stage −3), subclinical FOG (Stage −2), and the pre‐FOG state (Stage −1), thereby expanding the therapeutic window. Early identification of vulnerability and detection of subclinical and pre‐FOG states may enable interventions aimed at delaying or preventing the emergence of freezing. As in Figure 1, stages may overlap and fluctuate across contexts, and features of later stages can be elicited under sufficiently challenging conditions. This perspective emphasizes a shift from reactive to proactive management of FOG across stages. See Herman et al. 2023, Tosserams et al. 2025, and Tosserams et al. 2026 for possible therapeutic approaches in these different stages.

Empirical observations, such as those shown in Figure 2, where freezing can be elicited under challenging experimental conditions in FOG‐unaware individuals, as well as longitudinal findings on the prodromal predictors of FOG (Herman et al. 2023), support a broader shift toward earlier intervention. Figure 3 illustrates the contrast between traditional downstream management and emerging opportunities for upstream prevention, risk modification, and the building of resilience through harnessing compensatory networks. Conceptually, if interventions that enhance compensatory capacity can lengthen the time to transition from Stage −3 to Stage −1, it is plausible, though speculative, that they may also prolong the time to conversion from Stage −2 to Stage 0.

Emerging rehabilitation approaches that target locomotor, cognitive, and sensory integration networks are already recommended for intervention at earlier stages of freezing, including phases when it has not yet emerged, even if not explicitly framed within the current staging framework (Tosserams et al. 2025). Interventions such as cueing strategies, dual‐task training, and adaptability‐focused gait rehabilitation are designed to improve the very systems that appear to become unstable in the pre‐FOG and subclinical stages (Gilat et al. 2021; Cosentino et al. 2023). These approaches have been shown to improve freezing‐related gait disturbance, likely by enhancing compensatory control and reducing cognitive‐motor interference. “Upstream interventions” may thus widen the therapeutic window for FOG, as illustrated in Figure 3. However, direct evidence that such interventions prevent the onset of freezing remains limited at this point in time (Tosserams et al. 2025; Tosserams et al. 2026).

Evolving digital monitoring techniques may also contribute to an upstream approach through risk stratification and early identification in the prodromal (Stage −3) and subclinical (Stage −2) stages, as these methods can capture subtle changes beyond the clinical eye (Mancini, Afshari, et al. 2025). A complementary prevention‐focused framework likewise emphasizes identifying people with PD at heightened risk before clinical FOG emerges and testing interventions earlier in the disease course (Tosserams et al. 2026). As stated above, this perspective also reframes how so‐called “false positive” detections in real‐world monitoring studies may, in some cases, reflect early or subclinical expressions of freezing vulnerability. In this regard, the development and validation of sensitive protocols designed to elicit and quantify FOG will help to detect and characterize subclinical freezing (Zoetewei, Herman, et al. 2024; Howe et al. 2026; Ehgoetz Martens et al., n.d.‐b), thereby improving the alignment between experimental and real‐world observations.

As freezing may not follow a single linear pathway from prodromal vulnerability to overt episodes, but instead may entail multiple pathophysiological routes, sensor‐based detection of signals from multiple physiological sources is likely needed (Mancini, McKay, et al. 2025). Individuals may also differ in the rate at which they progress across stages, the extent to which they fluctuate between states, and which systems are most prominently involved at any given time. Integrating this heterogeneity into models of freezing may improve both prediction and interpretation and suggests that future work should move beyond stage‐based classification alone toward more individualized characterization (recall Tables 2, 3A, and B).

At the same time, this framework raises new challenges. Although certain clinical, gait, digital, and physiological measures are putatively associated with and may help distinguish the proposed stages (Tables 1 and 2; Figures 1 and 2), the boundaries between stages and the mechanisms governing transitions remain poorly defined. For example, we do not yet know why or when a person with PD moves from a vulnerable state in which FOG cannot be elicited (Stage −3) to one in which FOG becomes detectable only during a sensitive or provocative protocol (Stage −2), or what determines when transient pre‐FOG instability (Stage −1) evolves into overt ineffective stepping (Stage 0). As more precise definitions of the core of FOG episodes and their accompanying abnormal stepping behaviour have only recently become available (Gilat et al. 2026), these transitions require improved measurement tools and longitudinal validation across diverse populations and contexts. Long‐term, preferably prospective, multimodal observational studies will be needed not only to characterize the upstream stages, but also to quantify their boundaries, identify the factors that drive movement between them, and determine whether those transitions are modifiable.

The framework also raises important questions about clinical translation, including when and how early‐stage findings should inform care. Advances in wearable sensing, digital mobility assessment, and multimodal data integration, including neurophysiological signals, provide new opportunities to study and potentially treat freezing in more naturalistic settings (O'Hanlon et al. 2024; Lewis et al. 2022; Zoetewei, Ginis, et al. 2024; Mancini, McKay, et al. 2025). Real‐time adaptation with adaptive‐DBS is promising (Fekri Azgomi et al. 2025; Klocke et al. 2025), although it is not yet clear exactly which stages such approaches will be able to target. Aligning these approaches with a temporally informed framework and awareness of inter‐individual variability may help shift the field toward earlier detection, improved mechanistic understanding, and ultimately prevention (Tosserams et al. 2026), consistent with upstream management.

## 7 Conclusions

Freezing of gait does not begin when the feet stop moving. Rather, emerging evidence suggests that it reflects several processes evolving across multiple time scales. Recognizing and characterizing these upstream stages may yield new insights into underlying mechanisms and create new opportunities for earlier intervention and, ultimately, for prevention.

## Author Contributions

**Jeffrey M. Hausdorff:** conceptualization, writing – review and editing, writing – original draft, funding acquisition. **Kaylena A. Ehgoetz Martens:** writing – review and editing, funding acquisition. **Moran Gilat:** funding acquisition, writing – review and editing. **Jorik Nonnekes:** funding acquisition, writing – review and editing. **Martina Mancini:** funding acquisition, writing – review and editing. **J. Lucas McKay:** funding acquisition, writing – review and editing. **Pieter Ginis:** writing – review and editing, funding acquisition. **Jamie L. Adams:** funding acquisition, writing – review and editing. **Jennifer R. Mammen:** funding acquisition, writing – review and editing. **Erika E. Howe:** writing – review and editing. **Simon J. G. Lewis:** funding acquisition, writing – review and editing. **Fay Horak:** funding acquisition, writing – review and editing. **Alice Nieuwboer:** conceptualization, writing – review and editing, funding acquisition.

## Funding

This work was funded in part by a grant from the Michael J Fox Foundation MJFF‐024177.

## Conflicts of Interest

J.H., K.A.E.M., M.G., J.N., M.M., J.L.M., J.L.A., J.R.M., E.H., S.J.G.L., F.H., A.N.: None. P.G. is an employee of Clario, part of Thermo Fisher Scientific, which manufactures and distributes the wearable OPAL sensors used for motor symptom measurement in Parkinson's disease.

## Data Availability

De‐identified data will be placed on a public access repository.

## References

[ejn70721-bib-0001] Adams, J. L. , K. Dinesh , C. W. Snyder , et al. 2021. “A Real‐World Study of Wearable Sensors in Parkinson's Disease.” Npj Parkinsons Disease 7: 106. 10.1038/s41531-021-00248-w. PMC8629990 34845224

[ejn70721-bib-0002] Amboni, M. , F. Stocchi , G. Abbruzzese , et al. 2015. “Prevalence and Associated Features of Self‐Reported Freezing of Gait in Parkinson Disease: The DEEP FOG Study.” Parkinsonism & Related Disorders 21, no. 6: 644–649. 10.1016/j.parkreldis.2015.03.028. 25899545

[ejn70721-bib-0003] Barbe, M. T. , L. Tonder , P. Krack , et al. 2020. “Deep Brain Stimulation for Freezing of Gait in Parkinson's Disease With Early Motor Complications.” Movement Disorders 35, no. 1: 82–90. 10.1002/mds.27892. 31755599

[ejn70721-bib-0004] Bardakan, M. M. , G. R. Fink , L. Zapparoli , G. Bottini , E. Paulesu , and P. H. Weiss . 2022. “Imaging the Neural Underpinnings of Freezing of Gait in Parkinson's Disease.” NeuroImage: Clinical 35: 103123. 10.1016/j.nicl.2022.103123. PMC9421505 35917720

[ejn70721-bib-0005] Bikias, T. , D. Iakovakis , S. Hadjidimitriou , V. Charisis , and L. J. Hadjileontiadis . 2021. “DeepFoG: An IMU‐Based Detection of Freezing of Gait Episodes in Parkinson's Disease Patients via Deep Learning.” Frontiers in Robotics and AI 8: 537384. 10.3389/frobt.2021.537384. PMC8185568 34113654

[ejn70721-bib-0006] Bohnen, N. I. , P. Kanel , Z. Zhou , et al. 2019. “Cholinergic System Changes of Falls and Freezing of Gait in Parkinson's Disease.” Annals of Neurology 85, no. 4: 538–549. 10.1002/ana.25430. PMC6450746 30720884

[ejn70721-bib-0007] Cao, Z. , A. R. John , H. T. Chen , et al. 2021. “Identification of EEG Dynamics During Freezing of Gait and Voluntary Stopping in Patients With Parkinson's Disease.” IEEE Transactions on Neural Systems and Rehabilitation Engineering 29: 1774–1783. 10.1109/TNSRE.2021.3107106. 34428144

[ejn70721-bib-0008] Cockx, H. , J. Nonnekes , B. R. Bloem , R. van Wezel , I. Cameron , and Y. Wang . 2023. “Dealing With the Heterogeneous Presentations of Freezing of Gait: How Reliable Are the Freezing Index and Heart Rate for Freezing Detection?” Journal of Neuroengineering and Rehabilitation 20, no. 1: 53. 10.1186/s12984-023-01175-y. PMC10134593 37106388

[ejn70721-bib-0009] Conde, C. I. , W. Puyati , D. M. Mijnarends , et al. 2024. “Triggers for Freezing of Gait in Individuals With Parkinson's Disease: A Systematic Review.” Frontiers in Neurology 14: 1328394. 10.3389/fneur.2023.1328394. PMC10771308 38187152

[ejn70721-bib-0010] Cosentino, C. , M. Putzolu , S. Mezzarobba , et al. 2023. “One Cue Does Not Fit All: A Systematic Review With Meta‐Analysis of the Effectiveness of Cueing on Freezing of Gait in Parkinson's Disease.” Neuroscience and Biobehavioral Reviews 150: 105189. 10.1016/j.neubiorev.2023.105189. 37086934

[ejn70721-bib-0011] Craig, C. E. , P. Kanel , N. J. Ray , and N. I. Bohnen . 2026. “Asymmetric Cholinergic Basal Forebrain Atrophy Marks Freezing of Gait in Parkinson's Disease.” European Journal of Neuroscience 63: e70467. 10.1111/ejn.70467. PMC13006747 41866810

[ejn70721-bib-0012] D'Cruz, N. , J. Seuthe , C. De Somer , et al. 2022. “Dual Task Turning in Place: A Reliable, Valid, and Responsive Outcome Measure of Freezing of Gait.” Movement Disorders 37, no. 2: 269–278. 10.1002/mds.28887. 34939224

[ejn70721-bib-0013] D'Cruz, N. , G. Vervoort , S. Chalavi , B. W. Dijkstra , M. Gilat , and A. Nieuwboer . 2021. “Thalamic Morphology Predicts the Onset of Freezing of Gait in Parkinson's Disease.” npj Parkinson's Disease 7, no. 1: 20. 10.1038/s41531-021-00163-0. PMC7925565 33654103

[ejn70721-bib-0014] D'Cruz, N. , G. Vervoort , S. Fieuws , C. Moreau , W. Vandenberghe , and A. Nieuwboer . 2020. “Repetitive Motor Control Deficits Most Consistent Predictors of Conversion to Freezing of Gait in Parkinson's Disease: A Prospective Cohort Study.” Journal of Parkinson's Disease 10, no. 2: 559–571. 10.3233/JPD-191759. 32039860

[ejn70721-bib-0015] Denk, D. , T. Herman , D. Zoetewei , et al. 2022. “Daily‐Living Freezing of Gait as Quantified Using Wearables in People With Parkinson Disease: Comparison to Self‐Report and Provocation Tests.” Physical Therapy 102: pzac129. 10.1093/ptj/pzac129. PMC10071496 36179090

[ejn70721-bib-0016] Economou, N. T. , J. L. McKay , D. Quek , et al. 2021. “Heart Rate Changes Prior to Freezing of Gait Episodes Are Related to Anxiety.” Journal of Parkinson's Disease 11, no. 1: 271–282. 10.3233/JPD-202146. 33074191

[ejn70721-bib-0017] Ehgoetz Martens, K. A. , et al. n.d.‐a. “Patient‐Reported Outcomes for Freezing of Gait: A Narrative Review and Consensus Recommendations by the FOG Clinical Outcomes Assessment Working Group.” Manuscript submitted for publication.

[ejn70721-bib-0018] Ehgoetz Martens, K. A. , J. M. Hall , M. J. Georgiades , et al. 2018. “The Functional Network Signature of Heterogeneity in Freezing of Gait.” Brain 141, no. 4: 1145–1160. 10.1093/brain/awy019. 29444207

[ejn70721-bib-0019] Ehgoetz Martens, K. A. , E. L. Lukasik , M. J. Georgiades , et al. 2018. “Predicting the Onset of Freezing of Gait: A Longitudinal Study.” Movement Disorders 33, no. 1: 128–135. 10.1002/mds.27208. 29150872

[ejn70721-bib-0020] Ehgoetz Martens, K. A. , D. S. Peterson , Q. J. Almeida , S. J. G. Lewis , J. M. Hausdorff , and A. Nieuwboer . 2020. “Behavioural Manifestations and Associated Non‐Motor Features of Freezing of Gait: A Narrative Review and Theoretical Framework.” Neuroscience and Biobehavioral Reviews 116: 350–364. 10.1016/j.neubiorev.2020.06.026. 32603716

[ejn70721-bib-0021] Ehgoetz Martens, K. A. , J. M. Shine , C. C. Walton , et al. 2018. “Evidence for Subtypes of Freezing of Gait in Parkinson's Disease.” Movement Disorders 33, no. 7: 1174–1178. 10.1002/mds.27417. 30153383

[ejn70721-bib-0022] Ehgoetz Martens, K. A. , et al. n.d.‐b. “Clinician‐Reported Outcomes for Assessing Freezing of Gait: A Call for Standardization and Consensus Recommendations by the FOG Clinical Outcomes Assessment Working Group.” Manuscript under review.

[ejn70721-bib-0023] Factor, S. A. , N. K. Steenland , D. S. Higgins , et al. 2011. “Postural Instability/Gait Disturbance in Parkinson's Disease Has Distinct Subtypes: An Exploratory Analysis.” Journal of Neurology, Neurosurgery, and Psychiatry 82, no. 5: 564–568. 10.1136/jnnp.2010.222042. PMC4646086 20884673

[ejn70721-bib-0024] Factor, S. A. , D. Weinshenker , and J. L. McKay . 2025. “A Possible Pathway to Freezing of Gait in Parkinson's Disease.” Journal of Parkinson's Disease 15, no. 2: 282–290. 10.1177/1877718X241308487. PMC13347428 39973500

[ejn70721-bib-0025] Faerman, M. V. , C. Cole , K. Van Ooteghem , et al. 2025. “Motor, Affective, Cognitive, and Perceptual Symptom Changes Over Time in Individuals With Parkinson's Disease Who Develop Freezing of Gait.” Journal of Neurology 272, no. 5: 321. 10.1007/s00415-025-13034-y. 40198411

[ejn70721-bib-0026] Fekri Azgomi, H. , K. H. Louie , J. E. Bath , et al. 2025. “Modeling and Optimizing Deep Brain Stimulation to Enhance Gait in Parkinson's Disease: Personalized Treatment With Neurophysiological Insights.” npj Parkinson's Disease 11, no. 1: 173. 10.1038/s41531-025-00990-5. PMC12177069 40533464

[ejn70721-bib-0027] Georgiades, M. J. , J. M. Shine , M. Gilat , et al. 2019. “Hitting the Brakes: Pathological Subthalamic Nucleus Activity in Parkinson's Disease Gait Freezing.” Brain 142, no. 12: 3906–3916. 10.1093/brain/awz325. 31665229

[ejn70721-bib-0028] Georgiades, M. J. , J. M. Shine , M. Gilat , et al. 2023. “Subthalamic Nucleus Activity During Cognitive Load and Gait Dysfunction in Parkinson's Disease.” Movement Disorders 38, no. 8: 1549–1554. 10.1002/mds.29455. PMC10946988 37226972

[ejn70721-bib-0029] Giladi, N. , M. P. McDermott , S. Fahn , et al. 2001. “Freezing of Gait in PD: Prospective Assessment in the DATATOP Cohort.” Neurology 56, no. 12: 1712–1721. 10.1212/WNL.56.12.1712. 11425939

[ejn70721-bib-0030] Giladi, N. , H. Shabtai , E. S. Simon , S. Biran , J. Tal , and A. D. Korczyn . 2000. “Construction of Freezing of Gait Questionnaire for Patients With Parkinsonism.” Parkinsonism & Related Disorders 6, no. 3: 165–170. 10.1016/S1353-8020(99)00062-0. 10817956

[ejn70721-bib-0031] Giladi, N. , J. Tal , T. Azulay , et al. 2009. “Validation of the Freezing of Gait Questionnaire in Patients With Parkinson's Disease.” Movement Disorders 24, no. 5: 655–661. 10.1002/mds.21745. 19127595

[ejn70721-bib-0032] Gilat, M. , P. Ginis , D. Zoetewei , et al. 2021. “A Systematic Review on Exercise and Training‐Based Interventions for Freezing of Gait in Parkinson's Disease.” npj Parkinson's Disease 7: 81. 10.1038/s41531-021-00224-4 PMC8433229 34508083

[ejn70721-bib-0033] Gilat, M. , J. Nonnekes , N. D'Cruz , et al. 2026. “An Updated Definition of Freezing of Gait.” Nature Reviews. Neurology 22, no. 3: 172–181. 10.1038/s41582-025-01179-3. 41513745

[ejn70721-bib-0034] Goris, M. , P. Ginis , C. Hansen , et al. 2025. “Is the Freezing Index a Valid Outcome to Assess Freezing of Gait During Turning in Parkinson's Disease?” Frontiers in Neurology 15: 1508800. 10.3389/fneur.2024.1508800. PMC11750649 39845938

[ejn70721-bib-0035] Handojoseno, A. M. , J. M. Shine , T. N. Nguyen , Y. Tran , S. J. Lewis , and H. T. Nguyen . 2013. “Using EEG Spatial Correlation, Cross Frequency Energy, and Wavelet Coefficients for the Prediction of Freezing of Gait in Parkinson's Disease Patients.” Annual International Conference of the IEEE Engineering in Medicine and Biology Society 2013: 4263–4266. 10.1109/EMBC.2013.6610487. 24110674

[ejn70721-bib-0036] Herman, T. , Y. Barer , M. Bitan , S. Sobol , N. Giladi , and J. M. Hausdorff . 2023. “A Meta‐Analysis Identifies Factors Predicting the Future Development of Freezing of Gait in Parkinson's Disease.” npj Parkinson's Disease 9, no. 1: 158. 10.1038/s41531-023-00600-2. PMC10696025 38049430

[ejn70721-bib-0037] Hoenig, M. C. , V. Dzialas , A. Drzezga , and T. van Eimeren . 2023. “The Concept of Motor Reserve in Parkinson's Disease: New Wine in Old Bottles?” Movement Disorders 38: 16–20. 10.1002/mds.29266. 36345092

[ejn70721-bib-0038] Howe, E. E. , P. Ginis , K. A. Ehgoetz Martens , et al. 2026. “The FOG‐COA Protocol: Validation of Freezing of Gait Clinical Outcomes Assessments in Parkinson's Disease.” European Journal of Neuroscience In press. 10.1111/ejn.70701 PMC13675721 42888898

[ejn70721-bib-0039] Hulzinga, F. , A. Nieuwboer , B. W. Dijkstra , et al. 2020. “The New Freezing of Gait Questionnaire: Unsuitable as an Outcome in Clinical Trials?” Movement Disorders Clinical Practice 7, no. 2: 199–205. 10.1002/mdc3.12893. PMC7011794 32071940

[ejn70721-bib-0040] Jansen, J. A. F. , T. T. C. Capato , S. K. L. Darweesh , et al. 2023. “Exploring the Levodopa‐Paradox of Freezing of Gait in Dopaminergic Medication‐Naive Parkinson's Disease Populations.” npj Parkinson's Disease 9, no. 1: 130. 10.1038/s41531-023-00575-0. PMC10492797 37689706

[ejn70721-bib-0041] Klocke, P. , M. A. Loeffler , S. J. G. Lewis , A. Gharabaghi , and D. Weiss . 2025. “Could Adaptive Deep Brain Stimulation Treat Freezing of Gait in Parkinson's Disease?” Journal of Neurology 272, no. 4: 267. 10.1007/s00415-025-13000-8. PMC11903562 40072634

[ejn70721-bib-0042] Klocke, P. , M. A. Loeffler , H. Muessler , M. S. Breu , A. Gharabaghi , and D. Weiss . 2024. “Supraspinal Contributions to Defective Antagonistic Inhibition and Freezing of Gait in Parkinson's Disease.” Brain 147, no. 12: 40–4071. 10.1093/brain/awae223 39470410

[ejn70721-bib-0043] Lewis, S. , S. Factor , N. Giladi , A. Nieuwboer , J. Nutt , and M. Hallett . 2022. “Stepping Up to Meet the Challenge of Freezing of Gait in Parkinson's Disease.” Translational Neurodegeneration 11, no. 1: 27. 10.1186/s40035-022-00298-x. PMC9057060 35490252

[ejn70721-bib-0044] Lewis, S. J. G. , and J. M. Shine . 2016. “The Next Step: A Common Neural Mechanism for Freezing of Gait.” Neuroscientist 22, no. 1: 72–82. 10.1177/1073858414559101. 25398230

[ejn70721-bib-0045] Loo, R. T. J. , L. Pavelka , G. Mangone , et al. 2025. “Interpretable Machine Learning for Cross‐Cohort Prediction of Motor Fluctuations in Parkinson's Disease.” Movement Disorders 40, no. 8: 1604–1617. 10.1002/mds.30223. PMC12371634 40843640

[ejn70721-bib-0046] Maidan, I. , M. Plotnik , A. Mirelman , A. Weiss , N. Giladi , and J. M. Hausdorff . 2010. “Heart Rate Changes During Freezing of Gait in Patients With Parkinson's Disease.” Movement Disorders 25: 2346–2354. 10.1002/mds.23280 PMC2964413 20721914

[ejn70721-bib-0047] Mancini, M. , M. Afshari , Q. Almeida , et al. 2025. “Digital Gait Biomarkers in Parkinson's Disease: Susceptibility/Risk, Progression, Response to Exercise, and Prognosis.” npj Parkinson's Disease 11, no. 1: 51. 10.1038/s41531-025-00897-1. PMC11928532 40118834

[ejn70721-bib-0048] Mancini, M. , N. Hasegawa , D. S. Peterson , F. B. Horak , and J. G. Nutt . 2023. “Digital Measures of Freezing of Gait Across the Spectrum of Normal, Non‐Freezers, Possible Freezers and Definite Freezers.” Journal of Neurology 270, no. 9: 4309–4317. 10.1007/s00415-023-11773-4. 37208526

[ejn70721-bib-0049] Mancini, M. , J. L. McKay , H. Cockx , et al. 2025. “Technology for Measuring Freezing of Gait: Current State of the Art and Recommendations.” Journal of Parkinson's Disease 15, no. 1: 19–40. 10.1177/1877718X241301065. PMC13347410 39973491

[ejn70721-bib-0050] Mancini, M. , V. V. Shah , S. Stuart , et al. 2021. “Measuring Freezing of Gait During Daily‐Life: An Open‐Source, Wearable Sensors Approach.” Journal of Neuroengineering and Rehabilitation 18, no. 1: 1. 10.1186/s12984-020-00774-3. PMC7784003 33397401

[ejn70721-bib-0051] Mazilu, S. , A. Calatroni , E. Gazit , A. Mirelman , J. M. Hausdorff , and G. Tröster . 2015. “Prediction of Freezing of Gait in Parkinson's From Physiological Wearables: An Exploratory Study.” IEEE Journal of Biomedical and Health Informatics 19, no. 6: 1843–1854. 10.1109/JBHI.2015.2465134. 26259206

[ejn70721-bib-0052] Mazilu, S. , A. Calatroni , E. Gazit , D. Roggen , J. M. Hausdorff , and G. Tröster . 2013. “Feature Learning for Detection and Prediction of Freezing of Gait in Parkinson's Disease.” In Machine Learning and Data Mining in Pattern Recognition, 144–158. Springer. 10.1007/978-3-642-39712-7_11.

[ejn70721-bib-0053] McMahon, N. E. , S. Visram , J. Popay , and M. Whitehead . 2022. “Framing Action to Reduce Health Inequalities: What Is Argued for Through Use of the ‘Upstream–Downstream’ Metaphor?” Journal of Public Health (Oxford, England) 44, no. 3: 671–683. 10.1093/pubmed/fdab099. PMC9424054 34056659

[ejn70721-bib-0054] Monaghan, A. S. , J. E. Gordon , S. Del Din , L. Rochester , and S. Lord . 2023. “Cognition and Freezing of Gait in Parkinson's Disease: A Systematic Review and Meta‐Analysis.” Neuroscience and Biobehavioral Reviews 147: 105068. 10.1016/j.neubiorev.2023.105068. 36738813

[ejn70721-bib-0055] Moore, A. , J. Li , C. H. Contag , et al. 2024. “Wearable Surface Electromyography System to Predict Freeze of Gait in Parkinson's Disease Patients.” Sensors (Basel) 24, no. 23: 7853. 10.3390/s24237853. PMC11644870 39686390

[ejn70721-bib-0056] Morris, T. R. , C. Cho , V. Dilda , et al. 2012. “A Comparison of Clinical and Objective Measures of Freezing of Gait in Parkinson's Disease.” Parkinsonism & Related Disorders 18, no. 5: 572–577. 10.1016/j.parkreldis.2012.03.001. 22445248

[ejn70721-bib-0057] Nieuwboer, A. , and N. Giladi . 2013. “Characterizing Freezing of Gait in Parkinson's Disease: Models of an Episodic Phenomenon.” Movement Disorders 28, no. 11: 1509–1519. 10.1002/mds.25683. 24132839

[ejn70721-bib-0058] Nieuwboer, A. , L. Rochester , T. Herman , et al. 2009. “Reliability of the New Freezing of Gait Questionnaire: Agreement Between Patients With Parkinson's Disease and Their Carers.” Gait & Posture 30, no. 4: 459–463. 10.1016/j.gaitpost.2009.07.108. 19660949

[ejn70721-bib-0059] Nutt, J. G. , B. R. Bloem , N. Giladi , M. Hallett , F. B. Horak , and A. Nieuwboer . 2011. “Freezing of Gait: Moving Forward on a Mysterious Clinical Phenomenon.” Lancet Neurology 10, no. 8: 734–744. 10.1016/S1474-4422(11)70143-0. PMC7293393 21777828

[ejn70721-bib-0060] O'Hanlon, C. E. , C. M. Farmer , J. Ryan , and N. Ernecoff . 2024. “Clinical Outcome Assessments and Digital Health Technologies Supporting Clinical Trial Endpoints in Early Parkinson's Disease: Roundtable Proceedings and Roadmap for Research.” RAND Health Quarterly 11, no. 3: 1. 10.7249/rhq-11-03-01 PMC11147643 38855392

[ejn70721-bib-0061] Palmerini, L. , L. Rocchi , S. Mellone , F. Valzania , and L. Chiari . 2017. “Identification of Characteristic Motor Patterns Preceding Freezing of Gait in Parkinson's Disease Using Wearable Sensors.” Frontiers in Neurology 8: 394. 10.3389/fneur.2017.00394. PMC5557770 28855887

[ejn70721-bib-0062] Pardoel, S. , A. AlAkhras , E. Jafari , J. Kofman , E. D. Lemaire , and J. Nantel . 2024. “Real‐Time Freezing of Gait Prediction and Detection in Parkinson's Disease.” Sensors (Basel) 24, no. 24: 8211. 10.3390/s24248211. PMC11679006 39771944

[ejn70721-bib-0063] Plotnik, M. , N. Giladi , and J. M. Hausdorff . 2012. “Is Freezing of Gait in Parkinson's Disease a Result of Multiple Gait Impairments? Implications for Treatment.” Parkinson's Disease 2012: 459321. 10.1155/2012/459321. PMC3263650 22288021

[ejn70721-bib-0064] Radojević, B. , N. T. Dragašević‐Mišković , A. Marjanović , et al. 2022. “The Correlation Between Genetic Factors and Freezing of Gait in Patients With Parkinson's Disease.” Parkinsonism & Related Disorders 98: 7–12. 10.1016/j.parkreldis.2022.03.018. 35398727

[ejn70721-bib-0065] Rose, G. 1985. “Sick Individuals and Sick Populations.” International Journal of Epidemiology 14, no. 1: 32–38. 10.1093/ije/14.1.32. 3872850

[ejn70721-bib-0066] Salomon, A. , E. Gazit , P. Ginis , et al. 2024. “A Machine Learning Contest Enhances Automated Freezing of Gait Detection and Reveals Time‐of‐Day Effects.” Nature Communications 15: 4853. 10.1038/s41467-024-49027-0. PMC11156937 38844449

[ejn70721-bib-0067] Schaafsma, J. D. , Y. Balash , T. Gurevich , A. L. Bartels , J. M. Hausdorff , and N. Giladi . 2003. “Characterization of Freezing of Gait Subtypes and the Response of Each to Levodopa in Parkinson's Disease.” European Journal of Neurology 10, no. 4: 391–398. 10.1046/j.1468-1331.2003.00611.x. 12823491

[ejn70721-bib-0068] Shalin, G. , S. Pardoel , E. D. Lemaire , J. Nantel , and J. Kofman . 2021. “Prediction and Detection of Freezing of Gait in Parkinson's Disease From Plantar Pressure Data Using Long Short‐Term Memory Neural Networks.” Journal of Neuroengineering and Rehabilitation 18, no. 1: 167. 10.1186/s12984-021-00958-5. PMC8626900 34838066

[ejn70721-bib-0069] Shine, J. M. , E. Matar , P. B. Ward , et al. 2013. “Freezing of Gait in Parkinson's Disease Is Associated With Functional Decoupling Between the Cognitive Control Network and the Basal Ganglia.” Brain 136: 3671–3681. 10.1093/brain/awt272. 24142148

[ejn70721-bib-0071] Sigcha, L. , D. Rodríguez‐Martín , N. Costa , et al. 2020. “Deep Learning Approaches for Detecting Freezing of Gait in Parkinson's Disease Patients Through On‐Body Acceleration Sensors.” Sensors (Basel) 20, no. 7: 1895. 10.3390/s20071895. PMC7181252 32235373

[ejn70721-bib-0072] Snijders, A. H. , C. A. Haaxma , Y. J. Hagen , M. Munneke , and B. R. Bloem . 2012. “Freezer or Non‐Freezer: Clinical Assessment of Freezing of Gait.” Parkinsonism & Related Disorders 18, no. 2: 149–154. 10.1016/j.parkreldis.2011.09.006. 21968033

[ejn70721-bib-0073] Sreenivasan, K. , E. Bayram , X. Zhuang , et al. 2023. “Topological Reorganization of Functional Hubs in Patients With Parkinson's Disease With Freezing of Gait.” Journal of Neuroimaging 33, no. 4: 547–557. 10.1111/jon.13107. PMC10523899 37080778

[ejn70721-bib-0074] Taylor, N. L. , G. Wainstein , D. Quek , S. J. G. Lewis , J. M. Shine , and K. A. Ehgoetz Martens . 2022. “The Contribution of Noradrenergic Activity to Anxiety‐Induced Freezing of Gait.” Movement Disorders 37, no. 7: 1432–1443. 10.1002/mds.28999. PMC9540856 35384055

[ejn70721-bib-0075] Tosserams, A. , A. Fasano , M. Gilat , et al. 2025. “Management of Freezing of Gait ‐ Mechanism‐Based Practical Recommendations.” Nature Reviews. Neurology 21, no. 6: 327–344. 10.1038/s41582-025-01079-6. 40169855

[ejn70721-bib-0076] Tosserams, A. , J. Nonnekes , B. R. Bloem , and E. M. Postma . 2026. “Towards the Prevention of Freezing of Gait in Parkinson's Disease.” European Journal of Neuroscience 64: e70636. 10.1111/ejn.70636. PMC13488605 42615931

[ejn70721-bib-0077] van Schalkwyk, M. C. I. , B. Hawkins , J. Collin , and M. Petticrew . 2026. “‘Who the Hell Is Upstream Pushing Them All in?’ Reclaiming Public Health's Defining Metaphor to Counter the Commercial Determinants of Health.” PLOS Global Public Health 6, no. 2: e0006045. 10.1371/journal.pgph.0006045. PMC12948116 41758808

[ejn70721-bib-0078] Virameteekul, S. , C. Shin , S. S. Hirczy , et al. 2026. “Assessing Digital Health Technologies for Outcome Measurement in Parkinson's Disease Drug Trials: A Systematic Review.” Movement Disorders 41, no. 1: 40–62. 10.1002/mds.70097. 41175008

[ejn70721-bib-0079] Walton, C. C. , J. M. Shine , J. M. Hall , et al. 2015. “The Major Impact of Freezing of Gait on Quality of Life in Parkinson's Disease.” Journal of Neurology 262, no. 1: 108–115. 10.1007/s00415-014-7524-3. 25319020

[ejn70721-bib-0080] Wang, D. , Y. Shi , J. Pang , X. Zhu , L. Meng , and D. Ming . 2026. “Data‐Driven Subtyping of Early Parkinson's Disease via Mutual Cross‐Attention Fusion of EEG and Dual‐Task Gait Features.” npj Parkinson's Disease 12, no. 1: 45. 10.1038/s41531-026-01258-2. PMC12905180 41526383

[ejn70721-bib-0081] Zhang, W. , S. S. Paul , J. D. Gwynn , L. Clemson , and L. F. Low . 2025. “Gait Characteristics in Older Adults With Higher‐Level Gait Disorders During Normal, Dual‐Task, and Destabilising Walking Conditions at Home and Their Association With Cognitive Impairment.” Gait & Posture 122: 286–291. 10.1016/j.gaitpost.2025.07.329. 40768791

[ejn70721-bib-0082] Zitser, J. , T. Herman , N. Giladi , and J. M. Hausdorff . 2025. “Waking up to the Role of Sleep Disturbances in Freezing of Gait Among People With Parkinson's Disease.” Parkinsonism & Related Disorders 133: 107342. 10.1016/j.parkreldis.2025.107342. 39988541

[ejn70721-bib-0083] Zoetewei, D. , P. Ginis , M. Goris , et al. 2024. “Which Gait Tasks Produce Reliable Outcome Measures of Freezing of Gait in Parkinson's Disease?” Journal of Parkinson's Disease 14, no. 6: 1163–1174. 10.3233/JPD-240134. PMC11380302 39121137

[ejn70721-bib-0084] Zoetewei, D. , P. Ginis , T. Herman , et al. 2025. “The Effects of Dopaminergic Medication and Task Load on Trembling and Akinetic Freezing of Gait in Parkinson's Disease.” Journal of Neurology 272, no. 4: 296. 10.1007/s00415-025-13023-1. 40153030

[ejn70721-bib-0085] Zoetewei, D. , T. Herman , P. Ginis , et al. 2024. “On‐Demand Cueing for Freezing of Gait in Parkinson's Disease: A Randomized Controlled Trial.” Movement Disorders 39, no. 5: 876–886. 10.1002/mds.29762. 38486430

